# Effectiveness of a Community-Based Crisis Resolution Team for Patients with Severe Mental Illness in Greece: A Prospective Observational Study

**DOI:** 10.1007/s10597-022-00983-1

**Published:** 2022-05-19

**Authors:** Aikaterini Koureta, Charalabos Papageorgiou, Charis Asimopoulos, Elisavet Bismbiki, Maria Grigoriadou, Stavroula Xidia, Theodora Papazafiri, Ilias I. Vlachos, Maria Margariti

**Affiliations:** 1 First Department of Psychiatry, Eginition Hospital, Medical School, National and Kapodistrian University of Athens, 11528 Athens, Greece; 2 Neurosciences and Precision Medicine Research Institute “Costas Stefanis”, University Mental Health, 115 27 Athens, Greece; 3 Department of Social work, University of West Attica, 12243 Athens, Greece; 4 Thriasio General Hospital of Elefsina, 19600 Elefsina, Greece; 5 General Hospital of Nikaia Agios Panteleimon, 18454 Peiraias, Greece; 6 Sismanogleio General Hospital, 15126 Athens, Greece; 7 Sotiria Hospital of Athens, 11527 Athens, Greece; 8 First Department of Psychiatry, Eginition Hospital, Medical School, National and Kapodistrian University of Athens, 72-74, Vassilisis Sofias str, 11528 Athens, Greece

**Keywords:** Crisis intervention, Psychiatric crisis, Community mental health, Hospitalization

## Abstract

This prospective observational study evaluated the effectiveness of a *crisis resolution team (*CRT) for outpatient treatment of psychiatric patients experiencing an acute episode of severe mental disorder. The effectiveness of the CRT (n = 65) was assessed against the *care-as-usual* [CAU group (n = 65)]. Patients’ clinical state, overall functioning, quality of life and satisfaction were respectively evaluated at baseline, post intervention and three-month post-intervention.

CRT patients compared to the CAU group, had significantly improved outcomes concerning clinical state and patient satisfaction at post intervention phase. Statistically significant improvement was also recorded for the dimensions of environment, physical and psychological health related to quality of life. No significant differences were observed between the two groups regarding overall functioning.

On the basis of these results, reforming of existing crisis-management services, in Greece, using the CRT model may improve substantially the services offered to psychiatric patients.

**Supplementary Information:**

The online version contains supplementary material available at 10.1007/s10597-022-00983-1.

## Introduction

For patients experiencing an acute psychiatric episode, hospital-based treatment is the standard procedure in most healthcare systems (Burns-Lynch et al., 2014; Cornelis et al., 2018). However, admission to psychiatric units has been associated with overall poor outcomes for patients such as loss of functionality and independence, lower recovery rate and stigmatization (Cornelis et al., 2018; Lieberman et al., 2017), delayed transition to community-based care, relapsing and higher rate of readmissions (Durbin et al., 2007; Tyler et al., 2019; Vigod et al., 2013). Concerns about the efficacy of hospital-based treatments have prompted clinicians and policy makers to seek alternative approaches for psychiatric crisis management for the treatment of patients with Severe Mental Illnesses (SMIs). Such an approach involves the treatment of psychiatric patients undergoing acute episodes as outpatients with the help of a Crisis Resolution Team (Johnson et al., 2005b).

The concept of Crisis Resolution Teams (CRTs) offering outpatient psychiatric services has been influenced by the crisis intervention theory and it is based on the assumption that acute psychiatric episode management is possible without hospitalization (Johnson, 2013; Lloyd-Evans et al., 2016). More specifically, CRTs are multidisciplinary teams, typically operating 24 h, 7 days per week, and may serve as an alternative to inpatient admission by offering emergency healthcare services and short-term treatment to patients in the community (Carpenter et al., 2013; Hasselberg et al., 2011). In recent decades, CRTs have been established as an integral part of the acute mental health system in several countries such as USA, Australia, Canada, U.K, Norway, Italy and other European countries (Hasselberg et al., 2011; Sjølie et al., 2010).

Despite their widespread use, CRT services aren’t clearly specified, leading often to variable forms of intervention while there is yet no adequate empirical evidence about their effectiveness (Cornelis et al., 2018; Lloyd-Evans et al., 2016; Stulz et al., 2020). Most studies on CRT’s effectiveness have elicited evidence concerning the limitation of hospital admissions (Glover et al., 2006; Jethwa et al., 2007; Johnson et al., 2005a) and cost-effectiveness Brown, 2005; Burns et al., 2001; Carpenter et al., 2013; Damsa et al., 2005; Hubbeling & Bertram, 2012; Mc Crone et al., 2009). However, research evidence concerning the effectiveness of CRTs with respect to clinical symptoms’ management, improvement of overall functionality and quality of life are yet limited. More specifically, existing data suggest that CRT-based treatments are associated with reduced psychiatric symptoms (Barker et al., 2011; Johnson et al., 2005b; Mötteli et al., 2018; Stulz et al., 2020) and improvement in overall functioning (Johnson et al., 2005a, 2005b; Mötteli et al., 2018; Stulz et al., 2020) compared to patients’ symptoms and functioning at admission, but there is yet no conclusive evidence favoring specifically the CRT over the care-as-usual *(*CAU) model. The only exception is the study of Mötteli et al., (2018) according to which scores on the Global Assessment of Functioning scale were significantly improved, at discharge, for Home Treatment patients compared to inpatients. Furthermore, previous research demonstrated that CRTs can increase patients’ satisfaction with acute care services and that the CRT model can be a more acceptable way of treating people with acute mental distress Barker et al., 2011; Carpenter et al., 2013; Johnson et al., 2005a, 2005b; Lloyd-Evans et al., 2016; Wheeler et al., 2015). Apart from these findings, there is no sufficient evidence of any further benefit of CRTs compared with CAU on patients’ quality of life (Johnson et al., 2005b).

Considering the burden of unnecessary hospital admissions on healthcare systems, investigation concerning the efficacy of CRT services for psychiatric acute episodes is essential. However, the operation of CRTs is highly complex and depends on a large number of variables. Consequently, exploring the relationship between CRT’s service features and outcomes is a methodological challenge. In response to the continuous increase of hospital admissions in Greece (Stylianidis et al., 2017) and according to the trends in psychiatric-crisis management worldwide, the 1st Department of Psychiatry of the National and Kapodistrian University of Athens (NKUA), at Eginition Hospital, has established a CRT focusing on psychiatric patients receiving crisis-management interventions. The specific CRT is a crisis management and resolution unit operating within the Emergency Department of the Psychiatric Clinic of Eginition Hospital, which operates 24 h a day, 7 days a week.

The objective of the present study was to examine the effectiveness of our CRT service for patients experiencing an acute mental illness episode, which would conventionally require hospital admission. Clinical symptoms, quality of life, overall functionality and service satisfaction were accessed. Data from patients who received CRT healthcare services was compared with data from patients who received conventional CAU treatment. Today, this CRT service, is the only standardized crisis management program for patients experiencing episodes in Greece that has been specifically designed to provide an alternative to hospitalization.

### Methodology

#### Design

The study was designed as a prospective observational study with the objective to evaluate the effectiveness of our CRT service for acute psychiatric episodes comparing to inpatient CAU. The study was conducted from September 2017 to September 2020 in five state hospitals (the Eginition hospital, the Sismanogleio General Hospital, the Sotiria Hospital of Athens, the General Hospital of Nikaia Agios Panteleimon, and the Thriasio General Hospital of Elefsina). The methodology of the study was in compliance with the Declaration of Helsinki, Good Clinical Practice guidelines, the General Data Protection Regulation and all the relevant National laws and regulations for conducting clinical research studies. Moreover, the protocol of the study was reviewed by the Ethics Review Board of each of the participating hospitals in order to receive informed consent prior to the initiation of the study. The study was also approved by the Scientific Review Board of the NKUA.

### Intervention Condition – CRT

The CRT service addresses adult outpatients who either present symptoms of acute psychopathology which require intensive psychiatric healthcare for the first time or to patients who experience exacerbation or relapse of an existing severe mental disorder that cannot be treated during routine psychiatric intervention and therefore require hospitalization. The main goals of our CRT service are the evaluation, treatment and stabilization of the acute psychopathological condition in outpatients and the prevention of hospitalization. The CRT is comprised of highly-trained, experienced professionals including a psychiatrist, who coordinates the team, a psychologist, a social worker, a psychiatric nurse and volunteer health professionals.

Key features of the CRT services include: (i) The provision of an individualized and patient-specific treatment plan, (ii) The provision of comprehensive psychiatric, psychological and social care based on the operation of a multidisciplinary team and on a participatory process, (iii) Emphasis on the principle of recovery (iv) Establishing cooperation with other services in the community to ensure therapeutic continuity and to support the recently introduced sectorization of mental health services across Greece’s National Health System (NHS). The treatment plan includes drug therapy, the patient’s psychotherapeutic support, family support, psychoeducation, referral and subsequent liaison with relevant mental health services in the community, in proximity to the patients’ residence.

The main condition for admittance to the CRT is the existence of a severe psychiatric episode that up to now was exclusively handled in the context of inpatient treatment unless these patients displayed increased potential to harm themselves or others. Patients who were primarily diagnosed with personality disorder or substance use disorder were excluded. Considering that CRT intervention is implemented in an outpatient context, cooperation between the CRT, the patient and his caregivers is favorable but not necessary in the case of patients who displayed cooperative behavior.

### Usual Care Condition

Standard care for psychiatric episodes which require hospital admission can be delivered with or without the consent of the patient. All healthcare services (pharmaceutical / psychiatric monitoring, psychosocial interventions, occupational therapy) are provided on a 24-hour daily basis. Professionals at a psychiatric ward are psychiatrists, medical doctors, psychologists, social workers, nurses and other mental health professionals.

### Participants

The participants of the study were the patients who collaborated with the CRT during the period September 2017 to September 2020 and the patients who were hospitalized during the same period at the collaborating hospitals. Patients evaluated by emergency clinicians as requiring hospitalization due to severe psychiatric crisis, were either admitted to CRT or to inpatient treatment depending on initial placement and the relevant criteria. In this paper, the term psychiatric crisis is defined as clinical symptoms severe enough to require formal admission to the inpatient psychiatric unit. The inclusion and exclusion criteria of the study were predominantly in accordance with the CRT’s inclusion and exclusion criteria. More specifically, for inclusion in the study, participants had to meet the following criteria: (1) experience of a psychiatric episode severe enough to require admission (2) age between 18 and 65 years old (3) access to some form of support outside the hospital and (4) no display of a tendency to harm others or themselves requiring inpatient monitoring. The exclusion criteria for participation in the study were as follows: (1) primary diagnosis of Axis I personality disorders or substance use disorders, according to the Diagnostic and Statistical Manual of Mental Disorders – 5th edition (DSM-V) (2) severe neurodevelopmental disorder and (3) referral for hospitalization following a court order. This final exclusion criterion was adopted as the majority of inpatients who display severe risk to harm themselves or others are usually involuntarily admitted to psychiatric wards and thus would skew the composition of the two groups towards more severe cases in the hospitalized group.

### Procedure

A total of 225 patients meeting eligibility criteria of the study, from the Eginition Hospital, where the CRT intervention was implemented, and from the collaborating hospitals which offer CAU treatment hospitals (the Sismanogleio General Hospital, the Sotiria Hospital of Athens, the General Hospital of Nikaia Agios Panteleimon, and the Thriasio General Hospital of Elefsina) were invited to participate in the study. Out of the initial 225 patients, 74 gave informed consent for the intervention group and 83 for the control group. Consent for participation in the study was sought during the first week of the patient’s contact with the participating hospitals. Patients were evaluated at baseline (prior to the inclusion in the study), at post intervention (at discharge) and at three-month post-intervention, a total of 130 patients (CRT group; n = 65 and CAU group; n = 65), all of whom completed the three phases of assessments and finally were all included in the study. Evaluation of the clinical status and overall functionality was conducted by two collaborating psychiatrists (mean scores were derived by consensus), while patients provided data on their quality of life and service satisfaction through self-reporting questionnaires.

### Data Collection Tools

#### Level of Functioning

The Global Assessment of Function Scale (GAF) was used to assess psychosocial functioning (Endicott et al., 1976). The GAF aims to evaluate the degree to which a person’s symptoms affect their daily life in terms of functionality, with no inclusion of the reduction of functionality due to physical or environmental limiting factors. The scale is divided into 9 sectors representing different levels of symptomatology and functionality. Each sector scores within a tenth of the total scale ranging from 1 to 10 to 81–90. Scores in the first tenth of the scale reflect severe clinical symptoms resulting in maximum reduction of functionality while the last tenth corresponds to satisfactory functionality and complete absence or minimal existence of symptoms. The scoring uses either the two codes at the two ends of each tenth or intermediate codes (Gotzamanis, 2004). GAF was administered in all phases (baseline, post intervention, 3-month post intervention).

### Psychiatric Symptoms

The clinical status has been evaluated using the Clinical Global Impression-Severity scale (CGI-S). The CGI-S is a 7-point scale that requires the clinician to assess the severity of the illness at the time of evaluation, based on previous experience with patients with the same diagnosis. Possible evaluations are: (1) Normal, (2) Marginally ill, (3) Mildly ill, (4) Moderately ill, (5) Visibly ill, (6) Seriously ill, (7) Severely ill (Busner & Targum, 2007). The score is based on symptoms observed and reported over the past seven days and reflects the average severity of the illness. The CGI-S scale was administered at baseline, post-intervention and three-month post-intervention.

### Quality of Life

To evaluate the quality of life, the World Health Organization Quality of Life (WHOQOL-BREF) questionnaire (WHO, 2004) was administered to all participants. WHOQOL is a self-report 26-item questionnaire rated on a five-point Likert scale, with four domains measuring: psychological health, physical health, social relationships and environment, plus 2 items representing the general Quality of Life (overall QOL and general health). Items 3, 4 and 26 of the questionnaire were negatively worded so their wording was reversed before the analysis so that higher scores represent higher QOL for all items. The Greek version includes a total of 30 items; 26 from the original English version and 4 from the validation and cultural adaptation of the questionnaire to the Greek population (Ginieri-Coccossis et al., 2012). Scoring for each item ranges from 1 to 5 (1 = not at all, 5 = completely). Internal consistency of Cronbach’s alpha for the Greek validation ranged from 0.67 to 0.81 while in the present study it ranged from 0.67 to 0.86 (baseline). WHOQOL-BREF was administered in all three phases of the study.

### Patient Satisfaction

A satisfaction questionnaire was developed by the authors, in order to explore specific aspects of satisfaction regarding services provided both to inpatients and outpatients. The questions addressed to the patients were about waiting time to join the program, awareness of the care they received, consideration of the patient in making decisions, feeling that the patient was treated with respect and as an equal, feeling that the care received was coordinated, satisfaction with the results of the treatment, consideration that the doctors took into account the difficulties in taking certain medications and dealing with the problem. Each item scored on a five-point Likert scale (1: not at all, 5: very much). For scale scoring, these eight items were summed and multiplied by 2.5 considering a maximum value of 100. Corrected item-total correlation values ranged from 0.31 to 0.77 and Cronbach’s alpha was 0.84. The patient satisfaction scale was administered post-intervention. In Table S1 there are presented all descriptive statistics of 8 items of the satisfaction questionnaire.

### The Interconnection Questionnaire

Sectorization of the Greek NHS while having been legislated is yet to be fully implemented. The interconnection questionnaire had two main goals. Firstly, the questionnaire aimed to investigate any difficulties in communication among collaborators and in consequently to estimate the impact of the forementioned incomplete process of sectorization on the therapeutic results of the two treatment modalities. Secondly, the questionnaire attempted to assess the readiness of services in responding to the multifaceted needs of patients. The interconnection questionnaire was completed by the researchers, via telephone, one month after the completion of the data collection process. It included 9 closed-ended questions.

### Statistical Analysis

Normally distributed variables are expressed as mean (M) and standard deviation (SD), while variables with skewed distribution are expressed as median (interquantile range). Qualitative variables were expressed as absolute and relative frequencies. For the comparison of proportions chi-square and Fisher’s exact tests were used. Students’*t*-test or Mann-Whitney test was used for the comparison of continuous variables between two groups. Repeated measurements analysis of variance (ANOVA) was adopted to evaluate the changes observed in investigated scales’ scores among the two study groups over the follow up period. Bonferroni correction was used in case of multiple testing in order to control for type I error. Logarithmic transformations were used at repeated measurements analysis of variance in case of CGI-S, due to non-normal distribution. All outcome variables were evaluated using completers only analysis. All reported *p*-values are two-tailed. Statistical significance was set at *p* < 0.05 and analyses were conducted using SPSS statistical software (IBM Corp. Released 2013. IBM SPSS Statistics for Windows, Version 22.0. Armonk, NY: IBM Corp.).

## Results

Sample characteristics are presented in Table 1. The majority of the patients in both study groups (CIP vs. CAU) were women (53.8% for both groups), Greeks (93.8% vs. 92.3% respectively), unmarried (56.9% vs. 52.3% respectively), without children (55.4% vs. 60.0% respectively), unemployed (53.8% vs. 61.5% respectively), who were living with others (76.9% for both groups). Mean age for patients in CIP group was 42.7 years (SD = 12.8 years) and for patients who were hospitalized (CAU group) was 46.8 years (SD = 13.5 years). For all of the aforesaid, there were non-significant differences between the two groups. Diagnosis (schizophrenic spectrum and emotional disorders) did not differ significantly between the two groups. The CRT group had a significantly greater frequency of psychotherapeutic support, family involvement and psychoeducation during the intervention. The average intervention time (difference between post- and baseline phase) was greater in the CRT group than the CAU group (with a median of 38 and 30 days respectively).

**Table 1:** Sample characteristics, by study group

		CRT (n = 65; 50%)	CAU (n = 65; 50%)	P
		n (%)	n (%)	
Gender				
	Men	30 (46.2)	30 (46.2)	> 0.999‡‡
	Women	35 (53.8)	35 (53.8)	
Nationality				
	Greek	61 (93.8)	60 (92.3)	> 0.999+
	Other	4 (6.2)	5 (7.7)	
Age, mean (SD)		42.7 (12.8)	46.8 (13.5)	0.075++
Years of education, mean (SD)		13.3 (3)	13 (4.1)	0.501++
Family status				
	Married	18 (27.7)	11 (16.9)	0.137+
	Unmarried	37 (56.9)	34 (52.3)	
	Separated	7 (10.8)	16 (24.6)	
	Widowed	3 (4.6)	4 (6.2)	
Children		29 (44.6)	26 (40.0)	0.594‡‡
Living alone		15 (23.1)	15 (23.1)	> 0.999‡
Working status				
	Employed	23 (35.4)	14 (21.5)	0.182‡‡
	Unemployed	35 (53.8)	40 (61.5)	
	Pensioner	7 (10.8)	11 (16.9)	
Average stay time (days), median (IQR) (difference post- vs. baseline)		38 (24 ─ 59)	30 (16 ─ 41)	**0.015**‡
Diagnosis				
	Schizophrenic spectrum	30 (46.2)	39 (60.0)	0.114‡‡
	Emotional disorders	35 (53.8)	26 (40.0)	
Psychiatric care				
	Yes	65 (100.0)	65 (100.0)	-
	No	0 (0.0)	0 (0.0)	
Psychotherapeutic support				
	Yes	41 (63.1)	30 (46.2)	**0.050**‡‡
	No	24 (36.9)	35 (53.8)	
Family intervention				
	Yes	56 (86.2)	35 (53.8)	**< 0.001**‡‡
	No	9 (13.8)	30 (46.2)	
Other support services				
	Yes	23 (35.4)	34 (52.3)	**0.050**‡‡
	No	42 (64.6)	31 (47.7)	
Psychoeducation				
	Yes	45 (69.2)	31 (47.7)	**0.013**‡‡
	No	20 (30.8)	34 (52.3)	

CRT; Crisis Resolution Team, CAU; care-as-usual, IQR; Interquartile range.

‡‡ Pearson’s chi square test; ‡Mann-Whitney test; +Fisher’s exact test; ++Student’s t-test.

Note. Significant differences are marked in bold.

Changes in patients’ CGI-S scores, GAF and quality of life scores throughout follow-up period, for each study group, are presented in Table 2. At baseline and three-month follow-up phase, there were no statistically significant differences between the two groups (CRT vs. CAU) for CGI-S (*p* = 0.758 and *p* = 0.991 respectively) in contrast with the post-intervention phase where the CAU group had clinical improvement (lowest CGI-S score) [M (SD); 2.26 (0.92) vs. 1.82 (0.66) respectively]. Both groups had significant differences between baseline, post-intervention and three-month follow-up phases (all *p* < 0.001). Evaluating the interaction of the group in the intervention phase, the CAU group had significantly less improvement in CGI-S compared with the CRT group (*p* < 0.001). Change between post-intervention vs. baseline, showed significant improvement of the clinical state in the CRT group [M (SD); -3.82 (1.01) vs. -3.35 (1.29) respectively]. However, this significant improvement in clinical state was not present at the three-month follow-up phase where non-significant differences between post-treatment vs. follow-up; *p* = 0.089 were observed.

**Table 2:** Changes in patients’ CGI-S, GAF and WHOQOL-BREF scores throughout follow-up period, by study group

		Phase 1	Phase 2	Phase 3	Change Phase 2 vs. 1	ChangePhase 3 vs. 2	Ρ^2^ Phase 1 vs. 2	Ρ^2^ Phase 2 vs. 3	Ρ^2^ Phase 1 vs. 3	Ρ^3^
		M (SD)	M (SD)	M (SD)	M (SD)	M (SD)				
CGI-S										
Clinical Global Impression for Severity	CRT	5.63 (0.91)	1.82 (0.66)	3.02 (1.51)	-3.82 (1.01)	1.20 (1.31)	**< 0.001**	**< 0.001**	**< 0.001**	**< 0.001**
	CAU	5.62 (1.07)	2.26 (0.92)	3.06 (1.68)	-3.35 (1.29)	0.80 (1.35)	**< 0.001**	**0.001**	**< 0.001**	
	P^1^	0.758	**0.001**	0.991	**0.025**	0.089				
GAF										
Global Assessment of Functioning	CRT	43.69 (11.48)	69.72 (13.18)	68.49 (13.99)	26.03 (13.95)	-1.23 (14.25)	**< 0.001**	> 0.999	**< 0.001**	0.369
	CAU	40.62 (15.36)	67.95 (14.87)	68.03 (19.25)	27.34 (16.46)	0.08 (12.04)	**< 0.001**	> 0.999	**< 0.001**	
	P^1^	0.198	0.474	0.876	0.626	0.573				
WHOQOL-BREF										
Overall Quality of Life & General Health	CRT	11.05 (3.88)	13.78 (3.16)	13.14 (3.69)	2.76 (3.76)	-0.65 (3.43)	**< 0.001**	0.252	**0.001**	0.514
	CAU	11.28 (4.37)	13.51 (3.08)	13.85 (2.99)	2.28 (4.20)	0.34 (3.33)	**< 0.001**	> 0.999	**< 0.001**	
	P^1^	0.751	0.656	0.224	0.493	0.099				
Physical Health	CRT	12.45 (2.97)	14.54 (2.36)	13.74 (3.03)	2.27 (3.10)	-0.72 (2.91)	**< 0.001**	**0.022**	**0.003**	**0.010**
	CAU	12.78 (3.64)	13.79 (2.56)	14.02 (2.25)	1.13 (3.30)	0.37 (2.23)	**0.038**	> 0.999	**0.004**	
	P^1^	0.503	0.156	0.354	**0.045**	**0.018**				
Psychological Health	CRT	10.49 (3.69)	13.26 (2.93)	12.58 (3.7)	2.80 (3.65)	-0.69 (3.26)	**< 0.001**	0.133	**< 0.001**	**0.020**
	CAU	11.24 (3.85)	12.85 (3.13)	13.03 (2.89)	1.61 (3.17)	0.48 (3.47)	**0.001**	> 0.999	**0.001**	
	P^1^	0.235	0.438	0.402	**0.050**	**0.049**				
Social Relationships	CRT	11.08 (3.55)	12.76 (3.5)	12.17 (3.34)	1.44 (3.88)	-0.57 (3.96)	**0.002**	> 0.999	0.069	0.902
	CAU	10.64 (3.64)	12.42 (3.12)	12.82 (3.09)	1.84 (3.94)	0.11 (3.21)	**< 0.001**	> 0.999	**< 0.001**	
	P^1^	0.460	0.987	0.342	0.564	0.279				
Environment	CRT	13.02 (2.54)	14.03 (2.37)	14.01 (2.2)	0.98 (2.23)	-0.02 (1.97)	**0.003**	> 0.999	**0.011**	0.375
	CAU	11.96 (2.84)	13.19 (2.29)	13.38 (2.32)	1.21 (2.45)	0.18 (2.13)	**< 0.001**	> 0.999	**< 0.001**	
	P^1^	**0.029**	**0.050**	0.117	0.590	0.565				

CGI-S; Clinical Global Impression-Severity scale, GAF; Global Assessment of Functioning Scale, WHOQOL-BREF; The World Health Organization Quality of Life, CRT; Crisis Resolution Team, CAU; care-as-usual, EDs; Emergency Departments, M; Mean, SD; Standard Deviation, Phase 1; pre-intervention phase, Phase 2; post-intervention phase, Phase 3; 3-month follow-up.

^1^p-value for group effect (Student’s *t*-test); ^2^p-value for time effect (Paired *t*-test); ^3^Effects reported include differences between the groups in the degree of change (mixed analysis of variance - ANOVA).

*Note.* For CGI-S analysis was conducted with logarithmic transformations. Significant differences are marked in bold.

In the three phases of the study, there were no statistically significant differences between the two groups (CRT vs. CAU) for the GAF scale. Moreover, concerning the GAF scale, both groups had significant differences between baseline and post-intervention phase as well as baseline and three-month follow up phase (all *p* < 0.001). There was no significant interaction of the group in intervention phases (*p =* 0.369). Changes between post-treatment vs. baseline and follow-up vs. post-treatment did not show significant differences for the two groups (*p* = 0.626 and *p* = 0.573 respectively).

In the three phases of the study, there were no statistically significant differences between the two groups (CRT vs. CAU) for all dimensions of the WHOQOL-BREF scale except dimensions concerning the Environment of the patient. The CRT group had higher scores than the CAU group for baseline-[M (SD); 13.02 (2.54) vs. 11.96 (2.84) respectively] and post- intervention phase [M (SD); 14.03 (2.37) vs. 13.19 (2.29) respectively]. For all WHOQOL-BREF dimensions, both groups had significant differences between baseline and post-intervention phase. Similarly, statistical findings were assessed between baseline phase and three-month follow up apart from the domain regarding Social Relationships in CRT group (*p* = 0.069). Comparing the post-intervention phase, the only difference that was identified was in the CRT group regarding Physical health (*p* = 0.022). Evaluating the interaction of the group across the three phases, the CRT group had significantly higher improvement in Physical and Psychological Health from baseline to follow-up phase (*p* = 0.010 and *p* = 0.020 respectively). For Physical and Psychological Health the same pattern was observed; the CRT group had significantly greater improvement between the 1st and 2nd phase compared to the CAU group, but the CAU group kept improving during phase 3 in contrast to the CRT group which worsened in performance on these two dimensions.

**Fig. 1:**
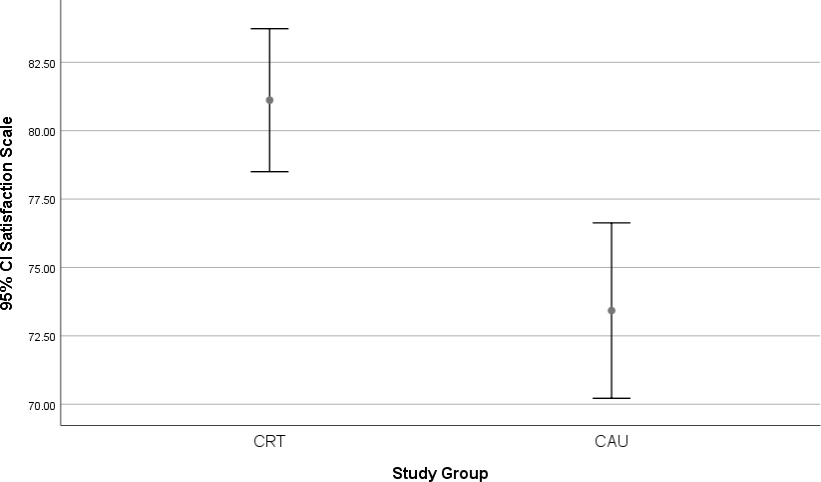
Error bar for Satisfaction Scale between study group. Abbreviations: CRT; Crisis Resolution Team, CAU; care-as-usual, CI; Confidence interval *P*-value for group effect (Student’s *t*-test) *Note* Significant differences with *p*-value < 0.001

Differences in the satisfaction scale between the study groups are presented in Fig. 1. Significantly higher scores (*p* < 0.001) were reported by the CRT group [M (SD); 81.12 (10.55)] in contrast with the CAU group [M (SD); 73.42 (12.94)].

Significant differences between the two groups were also detected with respect to the healthcare facility they were referred to (Table S2). More specifically, the patients who were hospitalized were mainly referred to the follow-up services of the same hospital, while those who participated in the CRT group were mainly referred to community mental health services in the vicinity of the patient’s residence. Also, the percentage of people in the CRT group who waited for more than a month for their first appointment with a psychiatrist was significantly higher than the CAU group (44.6% and 7.7% respectively). Similarly, the proportion of CRT patients who encountered difficulties in receiving healthcare services from the facility they were referred to for psychiatric follow-up was significantly higher than the CAU group (33.8% and 9.2% respectively). The rate of cooperation with the referral services was also significantly higher for the CAU group (72.3% vs. 50.8%). The majority of both groups collaborated with a service that belonged to the public sector. However, the participants of the CRT group eventually resorted to the private sector at a significantly higher rate compared to the CAU group (31.0% and 14.5% respectively). The frequency of those receiving psychotherapeutic support at the healthcare facility they were referred to after the crisis intervention was significantly higher among CAU group as well as the percentage of those receiving psychotherapeutic support from the institution that regularly monitored them. Participants in both groups desired to keep receiving psychotherapeutic services from the institutes they were regularly monitored by, with respect to their psychiatric condition.

## Discussion

The main objective of this prospective observational study was to evaluate the effectiveness of outpatient healthcare services provided by our CRT to patients experiencing acute psychiatric crises, as an alternative to hospitalization. To this end, the progress of patients who received CRT services was monitored comparing to patients who received standard hospital-based inpatient treatment (CAU). Patient’s monitoring was based on validated questionnaires (CGI-S, GAF, WHO-QOL) and a patients’ satisfaction questionnaire designed in the context of this study. Clinical outcome, level of functionality, quality of life and patient’s satisfaction were evaluated. Assessments were carried out over three phases: at baseline, at post intervention and at three-month post-intervention. In general, our findings confirmed our hypothesis that our CRT service is at least as effectiveness as the CAU model and that it is feasible to treat a significant percentage of psychiatric patients experiencing an acute episode in a community context without hospitalization. Overall, the results of the analysis showed that the effectiveness of CRT intervention differed from that of CAU in some respects, including severity of symptoms, satisfaction and some aspects of quality of life but not with respect to improvement of level of functioning.

Concerning clinical outcomes, patients assigned to the CRT group had significantly lower CGI-S scores in the post-intervention phase, corresponding to significant improvement in the severity of symptoms compared to the CAU group. This difference though, was not found to be significant between the two therapeutic models at the three-month follow-up phase. Evidence from two relatively recent systematic reviews on CRT effectiveness (Carpenter et al., 2013; Hubbeling & Bertram, 2012), do not support our findings as they have not identified significant differences concerning symptoms’ improvement between the two therapeutic models (CRT/CAU). Similarly, Johnson et al. in an observational study and a randomized controlled study (Johnson’s et al., 2005a, 2005b), report that CRT service users had similar symptomatic outcome comparing to inpatients at 6 and 8 weeks respectively, post-intervention. Concerning the short-term assessment of symptom’s’ severity based on the CGI-S scale, another recent observational study by Mötteli et al., (2018) also concluded that there were no significant differences on the CGI-S scores of psychiatric patients receiving home-treatment compared to hospitalized patients, at post intervention. Similar symptomatic outcomes were also recorded by Stulz et al., (2020) in their clinical trial comparing inpatients and outpatients with acute psychiatric episodes, although they used a different scale for symptoms’ assessment. Despite the discrepancy of our results for significant differences on the improvement in the severity of symptoms at post intervention between the two treatment modalities, the findings of this study concerning similar symptomatic outcomes in a long-term basis are in compliance with the aforementioned studies and reviews.

The findings of this study also indicated that both CRT and CAU patients had improved overall functionality on the GAF scale, post intervention and after the three-month follow-up assessment, compared to baseline scores. No differences though, were detected in the improvement of overall functionality between the two groups. Our findings are similar with relevant studies (Johnson et al., 2005a, 2005b; Stulz et al., 2020) which also demonstrated no statistically significant differences in overall functioning between the CRT and the CAU model of treatment with respect to patients’ functionality. On the other hand, Mötteli et al., (2018) recorded improved GAF scores for the patients receiving home-based treatment upon completion of their interventions comparing to hospitalized patients. It should be noted though that in that study (Mötteli et al., 2018), the participants potentially presented less severe symptoms due to affective disorders, while part of the sample was assigned to home treatment after receiving initial treatment as inpatients. Moreover, it should also be pointed out that the specific study does not include follow up assessments to confirm that these differences in functionality are long-standing. Interpreting overall functionality in a broader sense including social functioning, Carpenter et al., (2013) and Johnson et al., (2013) also claim that no significant differences between the two groups have been identified concerning the improvement of patients’ social abilities. Considering though that social functioning might also reflect more complex processes such as retaining employment or not becoming homeless, the overall period of these studies might be too short for the manifestation of such differences. Moreover, recent studies indicate that parameters such as initial severity of symptoms and certain demographic features (age, employment, family support) might play a far more definitive role in -functionality than the modality of treatment (Mötteli et al., 2021; Stulz et al., 2021).

Regarding outcomes with respect to Quality of life, statistically significant differences were detected in the items of the WHOQOL scale concerning the patients’ environment, at baseline and at the post intervention phase, favoring CRT patients. Similarly, significantly higher improvement of the physical and psychological health of patients assigned in the CRT group were detected between baseline and the post-intervention phase, while the CAU group demonstrated statistically significant improvement compared to the CRT group at the three-month follow-up evaluation. A previous relevant research study has not identified such differences between CRT and CAU groups (Johnson et al., 2005b), neither for short nor for long-term follow-up assessments, although there are studies suggesting that active treatment in the context of a community-based program, improves quality of life (Beecham et al., 2004; Lien, 2002). Moreover, considering that hospital admission carries a heavier stigma than receiving treatment within the community (Rose, 2001), the assumption that patients who avoid hospitalization might enjoy better quality of life with respect to their environment might not be farfetched. Actual data though, is yet inconclusive suggesting that further systematic research is required.

A more marked and consistent difference between the two groups of patients occurred with respect to treatment satisfaction. Participants assigned to the CRT group were significantly more satisfied by all measured aspects of the services they received compared to the CAU group. These findings are consistent with previous research data indicating that the CRT model may increase service users’ satisfaction Barker et al., 2011; Carpenter et al., 2013; Johnson, 2005a, 2005b; Ruggeri et al., 2006). A safe assumption could be that receiving psychiatric treatment in familiar surrounding rather than in a hospital environment, potentially constitutes a more positive experience (Winness et al., 2010) despite certain exceptions such as the study of Stulz et al., (2020) who identified no significant differences in patients’ satisfaction between the two treatment models.

Considering the validity of the major findings of this study regarding the prominence of our CRT service, at post intervention phase with respect to clinical outcome, quality of life and patients’ satisfaction, it could be argued that our CRT offers crisis management within a familiar environment based on a holistic approach, which may contribute significantly to better therapeutic results and more satisfactory relationships. In addition, the observed higher levels of satisfaction among CRT service users, can also be associated with better quality of life post intervention related to the environment, psychological and physical health when compared to CAU patients. However, these assumptions are questionable when looking at the results at three-month post intervention phase. What is evident is that improvement in clinical status and quality of life in CRT patients has no significant difference in comparison to CAU group after three-month post intervention. This irregularity could be more explained if we examine it in combination with the difficulties that have been recorded in the process of referral to the follow-up community mental health services because of the incomplete implementation of sectorization and the lack of a consistent framework for the provision of psychiatric healthcare in Greece. Therefore, as the majority of our CRT service users in contrast to CAU service users were referred to community mental health services, transition problems such as waiting-time for initial referral and change of therapeutic protocols were observed. The lack of stable psychiatric support due to extended referral waiting periods could have cause their regression. On the other hand, the majority of the CAU group remained for post-intervention treatment at the outpatient clinics of the institutes where they were hospitalized. Consequently, their transition to post-intervention treatment was smoother, as in several cases they even kept receiving treatment from the same healthcare professionals. This finding might be an indication that long term effectiveness of CRT treatment depends heavily on the quality of the mental health services of the respective community. Moreover, this finding of our study highlights the existing serious interconnection problems between patients and community mental health services and the inability of these services to respond to patient’s needs and requests.

Mirroring previous research data our findings suggest that the CRT model is more or equally effective with the CAU model with respect to clinical outcome, overall functioning, quality of life and patient’s satisfaction. However, it should be stressed that previous relevant studies mainly investigated the effectiveness of the CRT model in the prevention of hospital admissions as well as its cost-effectiveness whereas our study focused mainly on the evaluation of the CRT impact on clinical outcome, overall functioning, quality of life and service user’s satisfaction. Moreover, it should be noted that direct comparison of CRTs among international controlled trials is practically challenging, as the CRTs participating in various studies may differ widely in terms of structure, organization and provided services while the data collection tools and methods of analysis were significantly different.

The present study is the only prospective observational study conducted to investigate the efficacy of CRT interventions in Greece. The major strengths of our study are the simultaneous recruitment of both groups and a comprehensive and comparable measurement in terms of clinical and psychosocial outcomes between the two models of care (CRT/CAU). However, the methodology of the study also presented certain limitations. Firstly, our observational design was not based on random allocation or random sampling. Despite this noteworthy limitation, by including all CRT and CAU participants that matched with the inclusion and exclusion criteria of the study, allowed the analysis to reflect real world practice. Secondly, due to the lack of funding for the recruitment of independent researchers, the study was not blind in the assessments of participants. In addition, patients were aware of the treatment modality that they were cooperating with. As a result of these two factors some bias towards one of the treatment conditions cannot be ruled out. Another limitation was that authors did not investigate the type of disorder as an independent variable. A future suggestion is the investigation of any differences in all the estimated parameters regarding confounding factors such as the diagnosis. Finally, for the assessment of patient satisfaction a validated questionnaire was not used.

## Conclusions

The present study demonstrated that our CRT service appears to have noteworthy advantages compared to CAU treatment, concerning the improvement of overall clinical outcomes in patients experiencing acute psychiatric symptoms. Moreover, CRT treatment appears to elicit higher levels of patients’ satisfaction. Given the constant increase in psychiatric morbidity within the Greek population, as a consequence of the recent economic crisis (Economou et al., 2016; Kentikelenis et al., 2014; Pikouli et al., 2019) and the ongoing Covid-19 pandemic (Fountoulakis et al., 2021), the need for radical reforms in the national mental health system is imperative. Under these circumstances, we consider that the CRT services in Greece should be seriously considered for acute psychiatric episodes as a promising alternative to hospitalization. Towards this direction, further systematic research in this field of study is required.

## Electronic Supplementary Material

Below is the link to the electronic supplementary material.

Supplementary Material 1

## Data Availability

Future requests to access the datasets analyzed will be adjudicated by the Eginition Hospital management committee.
