# Supplementary material for: Effectiveness of a Community-Based Crisis Resolution Team for Patients with Severe Mental Illness in Greece: A Prospective Observational Study
Source: Community Ment Health J. 2022 May 19;59(1):14–24. doi: 10.1007/s10597-022-00983-1 (PMC9118182; doi:10.1007/s10597-022-00983-1)
Supplement: Supplementary file 1 — Supplementary Material 1 [file 10597_2022_983_MOESM1_ESM.docx]

Supplementary Tables

| Table S1.Descriptive statistics in the items of satisfaction questionnaire | | | | | |
| --- | --- | --- | --- | --- | --- |
|  | Not all *n* (%) | Slightly*n* (%) | Moderately*n* (%) | Very*n* (%) | Extremely*n* (%) |
| How long did you have to wait for your enrollment in the Program? / How long did you have to wait until the admission process to the hospital ward was completed? | 52 (40.3) | 52 (40.3) | 14 (10.9) | 6 (4.7) | 5 (3.9) |
| To what extent did you feel aware of the care you were receiving? | 0 (0.0) | 2 (1.5) | 8 (6.2) | 58 (44.6) | 62 (47.7) |
| To what extent did you consider yourself involved in decision-making about your care? | 2 (1.5) | 6 (4.6) | 24 (18.5) | 63 (48.5) | 35 (26.9) |
| To what extent did you feel treated with respect and equality? | 0 (0.0) | 1 (0.8) | 11 (8.5) | 56 (43.1) | 62 (47.7) |
| To what extent did you feel that the care you received was coordinated? | 0 (0.0) | 4 (3.1) | 13 (10.0) | 59 (45.4) | 54 (41.5) |
| How satisfied are you with the results of your treatment? | 0 (0.0) | 5 (3.8) | 14 (10.8) | 51 (39.2) | 60 (46.2) |
| To what extent did you consider that the doctors took into account your difficulties in taking certain medications? | 3 (2.3) | 5 (3.8) | 27 (20.8) | 60 (46.2) | 35 (26.9) |
| To what extent do you think that the CRT services/ Hospitalization helped you to deal with your problem? | 1 (1.4) | 5 (6.9) | 13 (18.1) | 25 (34.7) | 28 (38.9) |
|  | | | | | |

| Table S2.Differences in the items of interconnection questionnaire between two groups (CRT vs. CAU) | | | | |
| --- | --- | --- | --- | --- |
|  | | CRT | CAU | PPearson's χ^2^ test |
|  |  | n (%) | n (%) |  |
| Referral framework | To the same hospital | 24 (37.5) | 40 (61.5) | **0.004** |
|  | To other hospital | 6 (9.4) | 9 (13.8) |  |
|  | To CMHC | 29 (45.3) | 11 (16.9) |  |
|  | To NGO | 3 (4.7) | 5 (7.7) |  |
| The waiting time for your first appointment with a psychiatrist at the referral service for your regular psychiatric surveillance was over a month? | Yes | 29 (44.6) | 5 (7.7) | **<0.001** |
|  | No | 36 (55.4) | 60 (92.3) |  |
| Did you encounter difficulties in transitioning to the referral service? | Yes | 22 (33.8) | 6 (9.2) | **0.001** |
|  | No | 43 (66.2) | 59 (90.8) |  |
| Did you collaborate with the referral service? | Yes | 33 (50.8) | 47 (72.3) | **0.012** |
|  | No | 32 (49.2) | 18 (27.7) |  |
| If not, did you collaborate with another service? | Yes | 23 (71.9) | 14 (77.8) | 0.746+ |
|  | No | 9 (28.1) | 4 (22.2) |  |
| The service you were referred to belongs to: | Public sector | 42 (64.6) | 48 (73.8) | **0.027** |
|  | Private sector | 22 (33.8) | 11 (16.9) |  |
|  | NGO | 1 (1.7) | 6 (9.2) |  |
| Are you currently undergoing psychotherapy? | Yes | 10 (15.4) | 27 (41.5) | **0.001**+ |
|  | No | 55 (84.6) | 38 (58.5) |  |
| Is your family receiving therapeutic support? | Yes | 10 (15.4) | 15 (23.1) | 0.266 |
|  | No | 55 (84.6) | 50 (76.9) |  |
| Do you think it would be important for the above services to be offered by the same health provider? | Yes | 56 (86.2) | 54 (83.1) | 0.627 |
|  | No | 9 (13.8) | 11 (16.9) |  |
| CRT; Crisis Resolution Team, CAU; care-as-usual, CMHC; Community mental health center, NGO; non-governmental organization.*P*-value of Pearson’s χ^2^ test,^+^Fisher's exact test.*Note.*Significant differences are marked in bold. | | | | |
